# Schmerzmittelgebrauch in Deutschland: Eine Erhebung über die Jahre 2008 bis 2019

**DOI:** 10.1007/s00482-022-00661-x

**Published:** 2022-07-21

**Authors:** Hans-Christoph Diener, Walter Lehmacher, Elmar Kroth, Anette Lampert, Thomas Weiser

**Affiliations:** 1 Abteilung für Neuroepidemiologie, Institut für Medizinische Informatik, Biometrie und Epidemiologie (IMIBE), Medizinische Fakultät, Universität Duisburg-Essen, Hufelandstr 55, 45147 Essen, Deutschland; 2 Institut für Medizinische Statistik und Bioinformatik (IMSB), Universität zu Köln, Robert-Koch-Str. 10, Geb. 55, 50924 Köln, Deutschland; 3 Bundesverband der Arzneimittel-Hersteller e. V. (BAH), Ubierstr. 71–73, 53173 Bonn, Deutschland; 4 Sanofi-Aventis Deutschland GmbH, Industriepark Höchst, 65926 Frankfurt am Main, Deutschland

**Keywords:** Schmerzmittelgebrauch, OTC-Analgetika, Rx-Analgetika, Werbung, Analgesic use, OTC drugs, Prescription drugs, Advertisement

## Abstract

**Hintergrund:**

Publizierte Daten zum Pro-Kopf-Gebrauch von Schmerzmitteln in Deutschland liegen nur bis zum Jahr 2005 vor. In der vorliegenden Analyse wurden Daten für die Jahre 2008 bis 2019 ausgewertet. Es wurde der Gebrauch von rezeptpflichtigen wie rezeptfreien Analgetika untersucht, mögliche Einflussgrößen wurden diskutiert.

**Methodik:**

Der Pro-Kopf-Gebrauch von verschreibungspflichtigen und rezeptfreien Analgetika wurde auf Basis von verlässlichen Daten zu Rezepten und Bareinkäufen in Apotheken ermittelt (IMS Pharmascope®). Für rezeptfreie Präparate wurde zudem eine Auswertung nach Wirkstoffen vorgenommen. Zusätzlich wurden Werbeaufwendungen pharmazeutischer Hersteller und Veränderungen der Abgabewege als mögliche Einflussgrößen analysiert.

**Ergebnisse:**

Innerhalb des Beobachtungszeitraums ist der Gebrauch von verschreibungspflichtigen Analgetika angestiegen, während der Gebrauch von rezeptfreien Analgetika gesunken ist. Monopräparate machen den Großteil des Absatzes von rezeptfreien Schmerzmitteln aus. Der Anteil von Kombinationspräparaten war in den letzten Jahren rückläufig.

**Diskussion:**

Der Gebrauch von rezeptfreien Analgetika in Deutschland war zwischen 2008 und 2019 rückläufig. Damit setzt sich der Trend ab 1995, der in einer vorherigen Untersuchung beobachtet wurde, fort. Externe Faktoren wie der Anstieg von Werbeausgaben oder der leichtere Zugang über Versandapotheken scheinen den Gebrauch nicht zu beeinflussen.

**Zusatzmaterial online:**

Die Online-Version dieses Beitrags (10.1007/s00482-022-00661-x) enthält eine weitere Tabelle.

## Abstract (en)

**Background:**

There are previously published data on the per capita use of analgesics in Germany, but only to 2005. In the present analysis, data for the years 2008–2019 were evaluated. The use of prescription and nonprescription analgesics was investigated and possible influencing factors were discussed.

**Materials and methods:**

The per capita use of prescription and nonprescription analgesics was determined based on data on prescriptions and cash purchases in pharmacies (IMS Pharmascope®, Wiener Neudorf, Austria). Moreover, an evaluation according to active substances was performed for nonprescription drugs. In addition, pharmaceutical manufacturers’ advertising expenditures and changes in distribution channels were analyzed as possible influencing factors.

**Results:**

During the observation period 2008–2019, the use of prescription analgesics in Germany increased, while the use of nonprescription analgesics decreased. Single-agent drugs constitute the majority of sales of nonprescription analgesics. The share of combination drugs has decreased in recent years.

**Conclusion:**

The use of nonprescription analgesics in Germany decreased between 2008 and 2019. The trend as of 1995, which was observed in a previous investigation, is therefore continuing. External factors, such as the increase in advertising expenses or easier access via mail-order pharmacies, do not appear to influence use.

## Einleitung

Der Gebrauch von Schmerzmitteln wird ambivalent gesehen: Dem Nutzen für die Betroffenen stehen Bedenken vor einer zu häufigen Anwendung oder unerwünschten Arzneimittelwirkungen gegenüber. Dies betrifft in besonderem Maße den Gebrauch rezeptfreier Analgetika. Publizierte Daten zum Pro-Kopf-Gebrauch von Schmerzmitteln in Deutschland liegen bis 2005 vor. Die vorliegende Analyse knüpft daran an und ergänzt die Datenlage bis 2019. Darüber hinaus wurde untersucht, welche Faktoren den Gebrauch von rezeptfreien Schmerzmitteln beeinflussen könnten.

## Hintergrund und Fragestellung

Zwischen 1986 und 2005 lag der Pro-Kopf-Gebrauch von Schmerzmitteln in Deutschland auf einem relativ konstanten, im internationalen Vergleich niedrigen Niveau [1]. Demgegenüber steht eine Auswertung von zwei repräsentativen Bevölkerungsumfragen aus den Jahren 1998 sowie 2008 bis 2011, in der ein leichter Anstieg des Einsatzes von rezeptfreien Analgetika festgestellt wurde [2]. Nicht selten werden Befürchtungen geäußert, dass der Gebrauch von rezeptfreien Analgetika in Deutschland besorgniserregend hoch sei, beispielsweise im Kopfschmerzreport der TK [3]. In manchen Fachmedien finden sich sogar Forderungen nach einem Werbeverbot für rezeptfreie Schmerzmittel gegenüber Laien (z. B. [4]).

Ein Ziel der vorliegenden Analyse war es, die Entwicklung des Schmerzmittelgebrauchs über einen längeren Zeitraum (2008–2019) darzustellen und in den Kontext externer Faktoren zu stellen, um eine Diskussion auf der Basis valider empirischer Daten zu ermöglichen. Dabei wurde zwischen verschreibungspflichtigen (Rx) und rezeptfreien (OTC) Präparaten differenziert.

Weil sich das Verhalten von Patienten in erster Linie auf den Gebrauch von rezeptfreien Analgetika auswirkt, wurde in der vorliegenden Arbeit ein Fokus auf diese Arzneimittel gelegt. Zunächst erfolgte eine detaillierte Analyse des Gebrauchs von OTC-Analgetika nach Wirkstoffen. Im nächsten Schritt wurde untersucht, ob der Gebrauch von externen Faktoren wie Werbeaufwendungen abhängen könnte.

## Methodik

Als Basis für den Gebrauch und den Umsatz von Analgetika dienten die Daten aus dem IQVIA Pharmascope® National. Diese Daten erfassen die zu Lasten der gesetzlichen Krankenkassen abgerechneten Rezepte aus den Apothekenrechenzentren sowie Hochrechnungen von Privatrezepten und Barabgaben auf Basis von repräsentativen Apotheken- und Versandhandelspanels in Deutschland. Die Daten wurden durch die IQVIA Commercial GmbH & Co. OHG, Frankfurt am Main, erhoben. Es wurden die „Zähleinheiten“ (ZE) enteral anzuwendender Analgetika analysiert. Eine Zähleinheit entspricht z. B. einer Tablette oder 10 ml Saft oder Sirup.

Die Daten zu Werbeausgaben stammen von der GfK SE, Nürnberg (GfK OTC Mix). Es handelt sich hierbei um die Bruttoinvestitionen (vor Rabatten und Preisnachlässen). Bevölkerungsdaten wurden beim Statistischen Bundesamt abgerufen (Stichtag: 31.12. des jeweiligen Jahres). Daten zu Versandapotheken wurden einem Gutachten des IGES Instituts und des Deutschen Instituts für Wirtschaftsforschung e. V. (DIW Berlin) für die Bundesregierung entnommen [5].

Erfasst wurden Analgetika des ATC-Codes N02B („Andere Analgetika und Antipyretika“) und M01A („Nichtsteroidale Antiphlogistika und Antirheumatika“). Betäubungsmittel und Spezialpräparate für Migräne (z. B. Triptane) fallen nicht unter diese ATC-Codes und wurden nicht berücksichtigt. Für OTC-Analgetika wurde eine detailliertere Analyse nach Wirkstoffen bzw. Wirkstoffkombinationen durchgeführt, wie sie im IQVIA Pharmascope® National angegeben sind.

Zur Ermittlung von Korrelationen wurden die Daten als x/y-Punktewolken dargestellt, lineare Regressionen durchgeführt und geprüft, ob die Steigungen (b) von 0 verschieden sind. Die Ergebnisse für die Steigung werden mit 95 %-Konfidenzintervallen (95 %-KI) und *P*-Werten angegeben. Auswertung und Darstellung der Daten erfolgte mit Graphpad Prism (Version 8.0.2).

## Ergebnisse

### Pro-Kopf-Gebrauch

**Figure 1:**
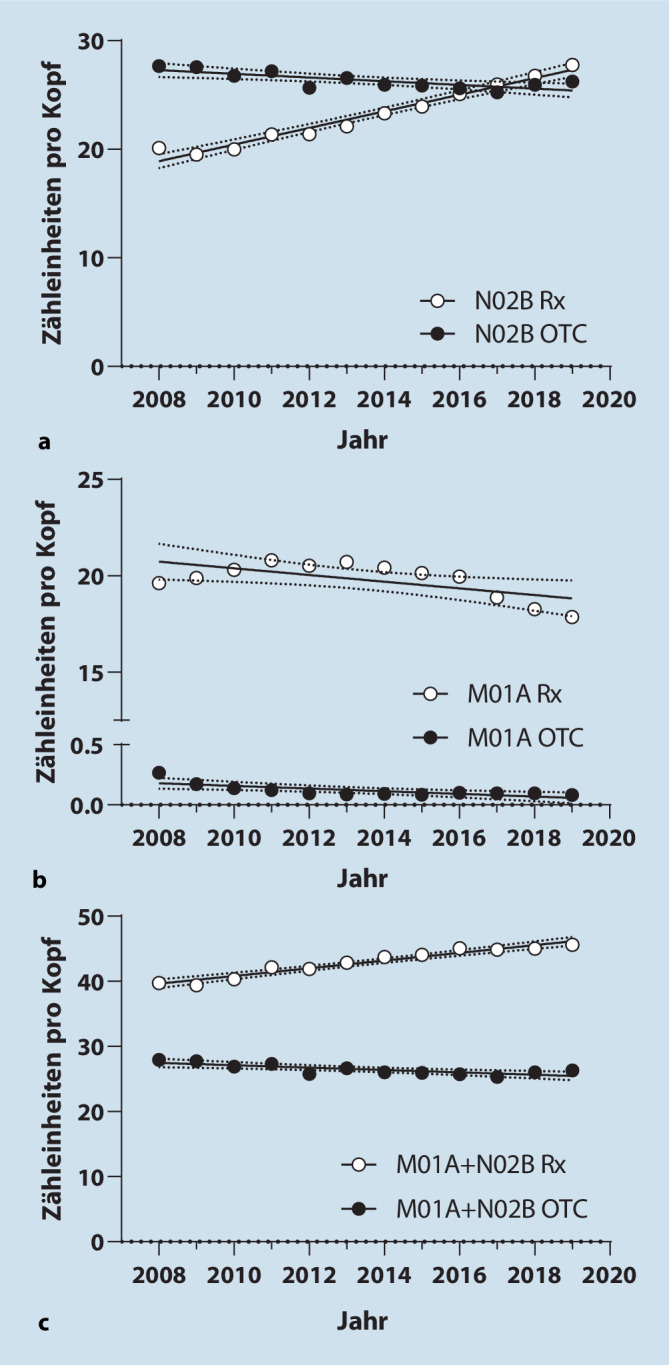

Insgesamt kam es zu einem leichten Anstieg des Pro-Kopf-Gebrauchs rezeptpflichtiger und rezeptfreier N02B-Analgetika von 48 ZE im Jahr 2008 auf 54 ZE im Jahr 2019, wohingegen der Gebrauch von M01A-Analgetika von 20 ZE auf 18 ZE abnahm. Bei den N02B-Analgetika nahm der Gebrauch von Rx-Präparaten zu (b = 0,76 ZE/Kopf * Jahr; 95 %-KI = 0,67 bis 0,86; *p* = 0,003; Abb. 1a); der Gebrauch von OTC-Präparaten ging dagegen zurück (b = −0,8 ZE/Kopf * Jahr; 95 %-KI = −0,27 bis −0,07; *p* = 0,003; Abb. 1a). Der Gebrauch bei den M01A-Analgetika nahm sowohl bei rezeptpflichtigen (b = −0,17 ZE/Kopf * Jahr; 95 %-KI = −0,32 bis −0,03; *p* = 0,0221) als auch bei rezeptfreien Präparaten ab (b = −0,01 ZE/Kopf * Jahr; 95 %-KI = −0,02 bis −0,004; *p* = 0,0053; Abb. 1b; Tabelle im Online-Zusatzmaterial). Insgesamt (N02B + M01A) ist der Gebrauch von Rx-Analgetika im Beobachtungszeitraum angestiegen (b = 0,59 ZE/Kopf * Jahr; 95 %-KI = 0,49 bis 0,69; *p* < 0,0001), während der Gebrauch von OTC-Analgetika gesunken ist (b = −0,18 ZE/Kopf * Jahr; 95 %-KI = −0,28 bis −0,08; *p* = 0,0025; Abb. 1c). Seit 2016 übersteigt der Pro-Kopf-Gebrauch von rezeptpflichtigen N02B-Analgetika den von rezeptfreien. Auch wenn man M01A- und N02B-Analgetika zusammen betrachtet, lag der Gebrauch rezeptpflichtiger Präparate im gesamten Beobachtungszeitraum über dem für rezeptfreie.

### Wirkstoffe in OTC-Analgetika

**Figure 2:**
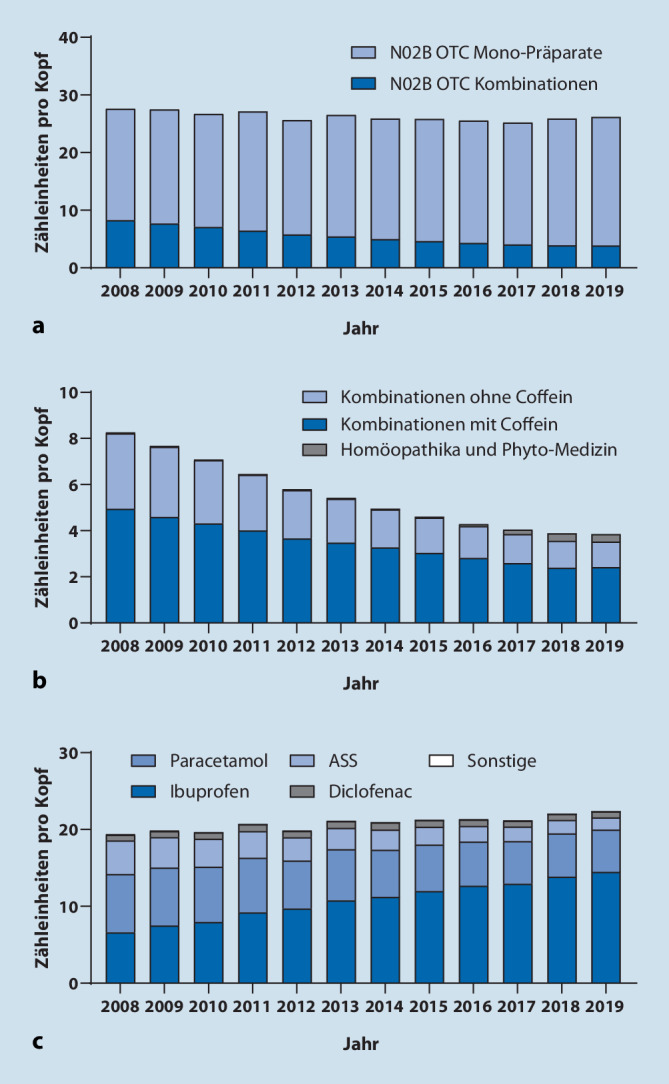

Bei den M01A-Analgetika machen OTC-Präparate einen sehr geringen Teil des Gesamtgebrauchs aus (Abb. 1b; Tabelle im Online-Zusatzmaterial), weshalb hier – anders als bei den N02B-Analgetika – keine Analyse nach Wirkstoffen vorgenommen wurde. Bei den N02B-Analgetika machen Monopräparate den Großteil des Absatzes von OTC-Analgetika aus. Kombinationsanalgetika haben einen relativ kleinen Anteil, der über den Beobachtungszeitraum weiter zurückgegangen ist (von 8,26 auf 3,84 ZE; Abb. 2a). Dies trifft gleichermaßen auf Kombinationen mit und ohne Coffein zu; homöopathische und Phytopräparate sind auf niedrigem Niveau angestiegen (Abb. 2b). Bei der Analyse nach Wirkstoffen wurde eine Zunahme des Gebrauchs von Ibuprofen festgestellt. Da der Gebrauch allgemein zurückgegangen ist, haben sich die Anteile der anderen Wirkstoffe bzw. Wirkstoffkombinationen entsprechend verringert (Abb. 2c; Tabelle im Online-Zusatzmaterial).

### Werbeaufwand

**Figure 3:**
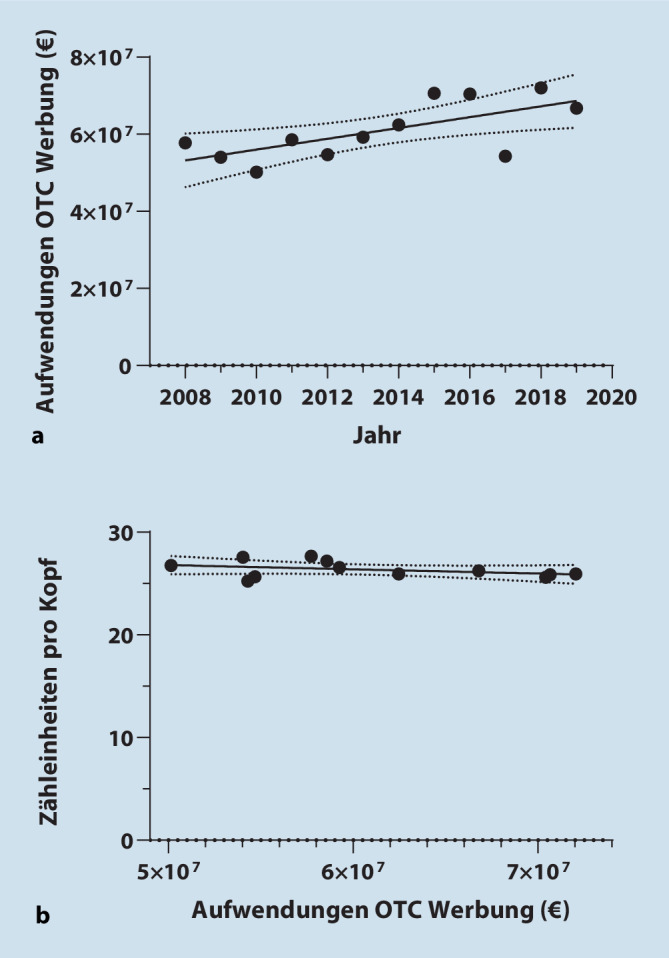

Der Bruttowerbeaufwand (ohne Rabatte und Preisnachlässe) für OTC-Analgetika ist von ca. 57 Mio. € im Jahr 2008 auf 67 Mio. € im Jahr 2019 gestiegen (Abb. 3a). Dieser Anstieg korreliert jedoch nicht mit dem Schmerzmittelgebrauch (b = −4,11^−8^ ZE/€ * Jahr; 95 %-KI = −1,10^−7^ bis 2,802^−8^; *p* = 0,21; Abb. 3b).

## Diskussion

### Pro-Kopf-Gebrauch

Die vorliegende Analyse zeigt, dass der Gebrauch von Schmerzmitteln zwischen 2008 und 2019 anstieg, was auf einen höheren Gebrauch von Rx-Analgetika zurückzuführen ist. Der Gebrauch von OTC-Analgetika ist dahingegen zurückgegangen. Das ist insbesondere hinsichtlich der manchmal gegenteiligen Darstellung in Laien- und Fachmedien bemerkenswert [3, 4]. Dieser Trend wurde bereits in einer Analyse des Schmerzmittelgebrauchs in Deutschland zwischen 1986 und 2005 beobachtet [1] und scheint sich damit fortzusetzen. Zusammengefasst (OTC und Rx) liegt der Gebrauch von N02B-Analgetika nach wie vor auf dem gleichen Niveau wie seit mehreren Jahren, sodass hier kein bzw. nur ein geringer Anstieg des Gesamtgebrauchs festzustellen ist.

Zur Ermittlung des Gebrauchs von Analgetika werden unterschiedliche Methoden angewendet. So erlauben beispielsweise Daten aus Befragungen die Beantwortung sehr spezifischer Fragen im Zusammenhang mit der Arzneimittelnutzung (zu Demografie, Anwendungsanlässen, Häufigkeit, Dosierungen etc.; siehe z. B. Sarganas et al. 2015 [2], Lange et al. 2014 [6]), unterliegen allerdings Störgrößen wie Erinnerungsverzerrungen („recall bias“) oder Berichterstattungsfehlern („reporting bias“).

In dieser Untersuchung wurde ein anderer Ansatz gewählt, der sich bereits früher bewährt hat [1], nämlich die Analyse auf Basis der in Deutschland abgegebenen „Zähleinheiten“ (ZE) enteral zu verabreichender Analgetika. Die Daten sind valide, da der Datenanbieter auf Basis von Krankenversicherungsdaten und Hochrechnungen aus einem relevanten Anteil von stationären und Versandapotheken in Deutschland präzise Angaben zur Abgabe machen kann. Diese Daten finden unter anderem Eingang in Gutachten, die für offizielle Stellen erarbeitet werden [5, 7].

Die Daten werden kontinuierlich erhoben und erlauben daher das Erstellen und Analysieren von Zeitreihen. Die lineare Regression über den hier beschriebenen (begrenzten) Zeitraum erlaubt die quantitative Beschreibung von Trends. Eine Extrapolation auf längere Zeiträume wäre nicht sinnvoll, da lineare Zusammenhänge darüber hinaus vermutlich nicht mehr gegeben sind.

### Wirkstoffe in OTC-Analgetika

Die Analyse der OTC-Analgetika nach Wirkstoffen zeigt, dass Ibuprofen den größten Anteil unter den OTC-Analgetika hat, der im Beobachtungszeitraum weiter zugenommen hat. Obwohl Ibuprofen ein effektiver und – insbesondere bei kurzzeitiger Anwendung im Rahmen der Selbstmedikation – gut verträglicher Wirkstoff ist, erzielte er in klinischen Studien nur bei rund jedem zweiten Patienten eine relevante Linderung von akuten Schmerzen [8]. Das Ansprechen auf Analgetika ist individuell verschieden [9]. Um allen Patienten wirksame Analgetika zur Verfügung stellen zu können, ist daher eine breite Palette an unterschiedlichen Präparaten wünschenswert. Das umfasst sowohl Monopräparate als auch Kombinationsanalgetika, die zum Teil höhere Therapieerfolge als Einzelwirkstoffe erzielen [8].

### Was beeinflusst den Schmerzmittelgebrauch?

Die vorliegende Analyse zeigt, dass im Beobachtungszeitraum der Pro-Kopf-Gebrauch rezeptpflichtiger Analgetika zugenommen hat. Der Anstieg des Analgetikagebrauchs steht im Einklang mit dem Anstieg des Anteils älterer Menschen in der Gesellschaft, da z. B. die Prävalenz von Erkrankungen und Schmerzen des muskuloskeletalen Systems mit dem Lebensalter korreliert sind [10]. Werbung und Preise entfallen als mögliche Faktoren, die den Gebrauch beeinflussen könnten, da Werbung nur für Fachkreise erlaubt ist und die Kosten in der Regel von der Krankenversicherung erstattet werden.

Anders als bei Rx-Medikamenten ist Laienwerbung für OTC-Arzneimittel zulässig. Patienten müssen rezeptfreie Arzneimittel in der Regel selbst bezahlen, und daher wäre eine Beeinflussung des Pro-Kopf-Gebrauchs sowohl durch Werbung als auch durch Preise denkbar. Eine Rolle könnte auch der bequeme Bezug über Versandapotheken spielen.

In dieser Analyse konnten keine Hinweise darauf gefunden werden, dass eine Erhöhung der Werbeausgaben zu einem höheren Pro-Kopf-Gebrauch von OTC-Analgetika führt. Das steht im Einklang mit der Annahme, dass pharmazeutische Hersteller in Analgetikawerbung investieren, um Marktanteile zulasten der Analgetika anderer Hersteller zu gewinnen – und nicht, um Menschen zur vermehrten Einnahme von Analgetika zu bewegen. Hierbei ist zu beachten, dass die Daten zu Werbeausgaben Bruttoinvestitionen (vor Rabatten bzw. Preisnachlässen) darstellen. Es ist daher davon auszugehen, dass die tatsächlichen Ausgaben für Werbung noch unter den hier angegebenen liegen.

Ein stetig wachsender Absatz von OTC-Arzneimitteln findet über Versandapotheken statt. Der Anteil am gesamten OTC-Abverkauf hat von 13 % im Jahr 2013 auf 19 % im Jahr 2019 zugenommen [5]; das heißt, diese niedrigschwellige Bezugsmöglichkeit für OTC-Präparate wird von Patienten zunehmend genutzt. In 2019 lagen die mittleren rabattierten Apothekenverkaufspreise in Vor-Ort-Apotheken bei −8,6 % und in Versandapotheken bei −27,6 % [5]. Insgesamt sind OTC-Arzneimittel in Versandapotheken also bequem zugänglich und kostengünstiger als in öffentlichen Apotheken. Doch auch der bequemere Bezug von OTC-Arzneimitteln zu niedrigeren Preisen induziert offenbar keinen Mehrgebrauch von Analgetika. In der Vergangenheit führte auch eine faktische Erhöhung der Preise für OTC-Arzneimittel (durch den Wegfall der Erstattungsfähigkeit zulasten der gesetzlichen Krankenkassen in 2004) nicht zu einer relevanten Beeinflussung des Gebrauchs von OTC-Analgetika (in 2000: 35,4 Analgetikadosen pro Kopf und Jahr, in 2005: 34,5; [1]).

In der vorliegenden Analyse konnten keine äußeren Faktoren identifiziert werden, die einen relevanten Einfluss auf den Gebrauch von OTC-Schmerzmitteln gehabt hätten. Das legt die Vermutung nahe, dass (OTC-)Analgetika tatsächlich deswegen eingenommen werden, weil die Anwender unter Schmerzen leiden. Diese Annahme wird auch durch die Ergebnisse einer Untersuchung des Robert Koch-Instituts gestützt: Hier zeigte sich ein hochsignifikanter Zusammenhang zwischen der Schwere der Schmerzen sowie der Schwere schmerzbedingter Einschränkungen und der Einnahme von OTC-Analgetika [6].

## Fazit für die Praxis

- Der Gebrauch von OTC-Analgetika ist in Deutschland zwischen 2008 und 2019 leicht zurückgegangen.
- Der Gebrauch von Rx- und OTC-Analgetika (N02B) zusammengenommen blieb relativ konstant und setzt damit den Trend der Jahre ab 1980 fort.
- Die Ergebnisse der vorliegenden Analyse legen nahe, dass externe Faktoren, wie der Anstieg von Werbeausgaben oder der leichtere Zugang durch Versandhandelsapotheken, den Gebrauch nicht erhöht haben.

### Supplementary Information





## Abkürzungen

ATC: Anatomisch-therapeutisch-chemische (Klassifikation)
b: Steigung
DIW: Deutsches Institut für Wirtschaftsforschung
KI: Konfidenzintervall
OTC: „Over the counter“, rezeptfreies (Arzneimittel)
Rx: Verschreibungspflichtiges (Arzneimittel)
ZE: Zähleinheiten
