# Erratum zu: Schmerzmittelgebrauch in Deutschland: Eine Erhebung über die Jahre 2008 bis 2019

**DOI:** 10.1007/s00482-022-00666-6

**Published:** 2022-08-12

**Authors:** Hans-Christoph Diener, Walter Lehmacher, Elmar Kroth, Anette Lampert, Thomas Weiser

**Affiliations:** 1 Abteilung für Neuroepidemiologie, Institut für Medizinische Informatik, Biometrie und Epidemiologie (IMIBE), Medizinische Fakultät, Universität Duisburg-Essen, Hufelandstr. 55, 45147 Essen, Deutschland; 2 Institut für Medizinische Statistik und Bioinformatik (IMSB), Universität zu Köln, Robert-Koch-Str. 10, Geb. 55, 50924 Köln, Deutschland; 3 Bundesverband der Arzneimittel-Hersteller e. V. (BAH), Ubierstr. 71–73, 53173 Bonn, Deutschland; 4 Sanofi-Aventis Deutschland GmbH, Industriepark Höchst, 65926 Frankfurt am Main, Deutschland

**Erratum zu:**

**Schmerz 2022**

10.1007/s00482-022-00661-x

Thomas Weiser wurde der Liste der Korrespondenzautoren hinzugefügt. Der Originalbeitrag wurde korrigiert.

