# Supplementary material for: Analgesic use in Germany: Survey of the years 2008 to 2019
Source: Schmerz. 2022 Jul 21;37(6):461–6. [Article in German] doi: 10.1007/s00482-022-00661-x (PMC10663189; doi:10.1007/s00482-022-00661-x)
Supplement: Supplementary file 1 [file 482_2022_661_MOESM1_ESM.pdf]

Zusatz-Tabelle

Jahr	M01A Rx	M01A OTC	N02B Rx	N02B OTC							
				Paracetamol	Ibuprofen	ASS	Diclofenac	Sonstige (z. B. Naproxen, Propyphenazon, Phenazon)	Kombinationen ohne Coffein	Kombinationen mit Coffein	Homöopathische und Phyto-Medizin
2008	19,61	0,27	20,10	7,57	6,62	4,37	0,75	0,09	3,28	4,94	0,04
2009	19,88	0,17	19,52	7,55	7,49	3,97	0,79	0,07	3,04	4,60	0,04
2010	20,30	0,14	19,99	7,21	7,93	3,64	0,82	0,06	2,74	4,31	0,04
2011	20,80	0,12	21,35	7,12	9,21	3,48	0,86	0,06	2,42	4,00	0,04
2012	20,52	0,10	21,38	6,24	9,71	3,04	0,83	0,06	2,09	3,66	0,04
2013	20,72	0,09	22,12	6,66	10,75	2,76	0,91	0,06	1,90	3,48	0,04
2014	20,42	0,09	23,32	6,14	11,22	2,63	0,92	0,05	1,64	3,27	0,04
2015	20,13	0,08	23,94	6,04	11,99	2,31	0,86	0,05	1,53	3,03	0,04
2016	19,95	0,10	25,09	5,75	12,66	2,06	0,81	0,05	1,38	2,81	0,09
2017	18,87	0,10	25,97	5,55	12,94	1,87	0,78	0,05	1,25	2,59	0,20
2018	18,27	0,10	26,77	5,64	13,83	1,76	0,77	0,05	1,17	2,39	0,33
2019	17,85	0,08	27,76	5,52	14,47	1,57	0,78	0,04	1,10	2,42	0,32

Pro-Kopf Gebrauch (in „Zähleinheiten“, ZU) von Analgetika des ATC-Codes M01A und N02B über die Jahre 2008 bis 2019. Angaben sind auf zwei Nachkommastellen gerundet. Datenbasis: IQVIA Pharmascope® National; Bevölkerungsdaten des Statistischen Bundesamtes.
